# Effect of Flushing Diets With Different Omega‐6 to Omega‐3 Fatty Acids Ratios on Reproductive Performance and Blood Biochemical Attributes in Shall Ewes

**DOI:** 10.1002/vms3.70775

**Published:** 2026-01-14

**Authors:** Mahmood Zeraatkar, Ahmad Riasi, Mohammad Ali Edriss, Javad Habibizad, Kimia Kazemi, Mohammad Choupani

**Affiliations:** ^1^ Department of Animal Sciences College of Agriculture, Isfahan University of Technology Isfahan Iran; ^2^ Department of Animal Sciences College of Agriculture, Yasouj University Yasouj Iran; ^3^ Department of Animal Sciences College of Agriculture, Ferdowsi University of Mashhad Mashhad Iran

**Keywords:** ewe, flushing, follicular growth, omega‐6: omega‐3 ratio, triglyceride and cholesterol, twinning rate

## Abstract

The aim of the present study was to evaluate effects of flushing diet supplemented with omega‐6 and omega‐3 sources on reproductive performance and blood biochemical parameters in ewes. Twenty‐five Shall ewes (30 ± 6 months) allocated to a completely randomized design with three experimental groups: (1) control diet with no fat supplementation (CON, *n* = 9), (2) control diet supplemented with calcium salt of soybean oil (CSSO, *n* = 8) and (3) diet supplemented with calcium salt of flaxseed oil (CSFO, *n* = 8). The ratio of omega‐6/omega‐3 in CON, CSSO and CSFO groups was 3.2, 5.3 and 1.5, respectively. The oestrous cycle of all ewes was synchronized with a progesterone‐impregnated sponge for a period of 13 days. From 4 days before to one day after removing sponges, the growth and activity of ovarian follicles were checked using transrectal ultrasound technique. Results showed supplementation diet with CSFO increased the number of large follicles near the time of sponge removal (*p* < 0.05). The CSFO group showed a significant (*p* < 0.05) increase in progesterone concentration on Day 12 post‐oestrus and improved oxidative status with decreasing (*p* < 0.05) in MDA and increasing (*p* = 0.06) TAC. The CSSO supplementation increased (*p* < 0.05) blood triglyceride and cholesterol levels. However, numerically higher prolificacy and fecundity were observed in the CSFO group. In conclusion, CSFO supplementation enhanced follicular growth and luteal function without affecting oestrus timing and reproductive outcome in Shall ewes.

## Graphical abstract

Dietary supplementation with different omega‐6:omega‐3 fatty acid ratios influenced ovarian activity, reproductive hormones and fertility outcomes in Shall ewes under semiarid conditions. Ewes receiving the lower ω6:ω3 ratio (1.5) showed improved follicular dynamics, higher progesterone levels and enhanced conception rate compared with higher ratios. These findings suggest that optimizing ω6:ω3 balance can support reproductive efficiency in ewes.

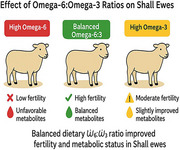

## 1 Introduction

The Shall sheep has good adaptation to adverse weather conditions. This Iranian large‐size and fat‐tailed sheep is primarily raised for meat production (Patiabadi et al. 2024; Hossein‐Zadeh 2015). It is well known that the reproductive efficiency of sheep is influenced by three factors: fertility, multiple births and lamb survival after birth. In fact, twinning is one of the most important characteristics of ewes that increases the income of livestock farmers (Scaramuzzi et al. 2006). Among the environmental factors affecting reproductive performance in sheep, feeding management has been reported to play a particularly significant role (Somchit‐Assavacheep 2011).

It has been shown that lipids play an essential role in mammalian reproduction by affecting the anterior pituitary, hypothalamus, ovaries and uterus. It seems that reproductive response depends on the type of fatty acids (FAs) consumption (Rahbar et al. 2014). Fat supplements can change the follicular dynamic and quality of corpus luteum by improving dietary energy, and synthesis of steroid hormones and eicosanoids (Rahbar et al. 2014; Zeron et al. 2002). Fat supplements may have different FA profiles and thus alter the ratio of omega‐6 to omega‐3 in diets (Moallem 2018). Mirzaei‐Alamouti et al. (2018) demonstrated that the FA content should be considered for evaluating the effect of fat supplements on livestock performance and their reproductive status. It is reported that farm animals can synthesize FA necessary for their proper health, except for FAs in the omega‐3 and omega‐6 families, which must be added to the diet (Moallem 2018). Consumption of omega‐3 FA had potential to facilitate membrane exchange by altering membrane fluidity and therefore affects the metabolism, growth and follicles development (Zachut et al. 2010). In a study conducted with goats, results showed that fish oil dietary supplementation increased the number of preovulatory follicles, ovulation rate and kidding rate (Mahla et al. 2017). In another study, Nieto et al. (2015) showed that consumption of fish oil and fish meal had positive effects on follicle growth and embryo development in sheep. Farrag et al. (2024) reported that calcium soaps of fatty acids (CSFAs) in diet of non‐pregnant ewes increased the number of ovarian follicles, especially large‐sized follicles (≥ 5 mm). Nurlatifah et al. (2022) showed that feeding ewes 2 weeks before and 2 weeks after mating with flushing rations containing 6% Lemuru fish oil (rich in omega‐3 FA) increased their twin births. It is determined that consumption of fats, improved the reproduction parameters through changes in the rumen fermentation pattern, increase the cholesterol synthesis, enhance steroid secretion from ovaries, changes in secretion of growth hormone and insulin and increase in IGF‐1 synthesis in ovarian cells (Rahbar et al. 2014).

There is limited information relating to the effect of flaxseed oil in pre‐mating diet on reproductive performance and serum metabolites of ewes (Didarkhah et al. 2020). We hypothesized that change the ratio of omega‐6/omega‐3 in flushing diet using the plant sources of omega FAs could increase the reproductive performance in Shall ewes. Therefore, the aim of present study was to investigate effect of flushing diets with varying omega‐6 to omega‐3 ratios on reproductive performance and selected blood biochemical parameters in Shall ewes.

## 2 Material and Methods

### 2.1 Animals and Experimental Procedures

This experiment was conducted from October to March (breeding season) 2024 at the Small Ruminant Research Center (SRRC) of Isfahan University of Technology, Isfahan, Iran (32°35′ N latitude and 52°33.5′ E longitude). In general, the climate of Isfahan is extremely dry and hot in summer season with an annual precipitation of < 125 mm.

Total of 25 oestrous cycling Shall ewes (2–3 years of age, 58 ± 5 kg BW) were randomly assigned to three experimental groups: (1) control diet with no fat supplementation (CON, *n* = 9, omega‐6:omega‐3 = 3.2), (2) diet supplemented with calcium salt of soybean oil (CSSO, *n* = 8, omega‐6:omega‐3 = 5.3) and (3) diet supplemented with calcium salt of flaxseed oil (CSFO, *n* = 8, omega‐6:omega‐3 = 1.5). To support our supplementation protocol (2% dietary DM), we followed the approach described by Farrag et al. (2024), who evaluated effects of daily supplementation with 30 g of calcium‐protected FAs on reproductive performance in Barki ewes. All ewes received the flushing diets for four consecutive weeks, including 4 weeks before sponge insertion and continuing until 1 day after sponge removal. Total duration of fat supplementation was 28 days. Flushing period follows standard field practices for inducing metabolic and hormonal changes prior to ovulation. This flushing window is widely used and has been shown to enhance ovarian response without imposing unnecessary costs on producers (Shad et al. 2011). All animals were tested for follicular activity using ultrasonography (4.5–8.5‐MHz frequency, Easi‐Scan ultrasound scanner, BCF Technology Ltd., Livingston, UK) and there was no previous evidence of reproductive or health problems. Three weeks before onset of the experimental treatments, all ewes were housed in individual pens (1.2 m × 2 m) with free access to water and maintenance diet. After adaptation, ewes fed flushing diets for 4 weeks. The diets were prepared as total mixed ration (TMR) and adjusted based on the requirements proposed in NRC (2001) for sheep. All diets were isocaloric and isonitrogenous (Table 1).

**TABLE 1:** The Ingredients and chemical composition and of experimental diets.

	Flushing diets^a^		
Ingredient (g/kg DM)	CON	CSSO	CSFO
Alfalfa hay	190	195	195
Wheat straw	349	390	390
Corn	288	190	190
Barley	90	100	100
Soybean meal	25	31	31
Wheat bran	40	60	60
Calcium salts of soybean oil	0	20	0
Calcium salts from flaxseed oil	0	0	20
Vitamin premix^b^	4	4	4
Mineral premix^c^	4	4	4
Calcium carbonate	5	1	1
Salt	5	5	5
Chemical composition, g/kg DM			
ME^d^, Mcal/kg of dry matter	2.2	2.2	2.2
Crude protein	104.3	104.8	105.3
Ether extract	21.6	25.5	24.4
Ash	97.3	104.1	104.2
Neutral detergent fibre	175.3	194.8	198.4
Fatty acids, g/kg			
Saturated fatty acids	612.8	598.2	595.7
Mono unsaturated fatty acids	255.7	263.1	260.8
Poly unsaturated fatty acids	52.7	61.6	72.3
Total omega‐6	26.3	30.8	22.3
Total omega‐3	8.2	5.8	14.8
Omega‐6:omega‐3 ratio	3.2	5.3	1.5

^a^ The control group (CON) without fat supplement and the other treatments supplemented with 2% of calcium salts of soybean oil (CSSO) and calcium salts of flaxseed oil (CSFO). Values are expressed in g/kg of diet; 20 g/kg corresponds to 2% of dietary DM.

^b^ Vitamin mix contained: vitamin A, 100,000 IU/kg; vitamin D3, 10,000 IU/kg; vitamin E, 8000 IU/kg.

^c^ Mineral mix contained: Co, 8 mg/kg [CoSO_4_]; Cu, 150 mg/kg [CuSO_4_]; I, 30 mg/kg[Ca(IO_3_)_2_]; Fe, 1000 mg/kg [FeSO4]; Mn, 1000 mg/kg [MnSO_4_]; Se, 6 mg/kg [Na_2_SeO_3_]; Zn, 2000 mg/kg [ZnSO_4_].

^d^ Metabolizable energy calculated according to NRC (2001).

The Ca‐salts of flaxseed oil and Ca‐salts of soybean oil kindly provided by Kimiya Danesh Alvand Co. (Tehran, Iran) contained 840 g/kg fat and 90 g/kg Ca as fed. The FAs composition of the feeds was analysed by extracting fatty acid methyl esters (FAME) as described by Hewavitharana et al. (2020). Hexane was utilized as a solvent for extraction and the extracted FAME was analysed with a gas chromatograph (Hewlett Packard, 5890, S.A., Barcelona, Spain). The carrier gas was helium with a constant flow of 1 mL/min, and the injector and detector were maintained at 240°C and 280°C, respectively. The oven temperature was held at 160°C for 5 min and then programmed to 210°C at 4°C/min, and held for 6 min at 210°C. The peaks of samples were identified and concentrations calculated based on the retention time and peak area of known standards. The amount of feed offered for each ewe was considered to be 1.2 kg/day during the maintenance period and 1.6 kg/day during the flushing program.

### 2.2 Synchronization of Oestrus and Mating

On Day 15th after the start of consuming experimental diets, the stage of oestrous cycle of all ewes was synchronized using of intravaginal progestin sponges (Medroxyprogesterone Acetate, Laboratories Hipra, S.A. Avda. La Selva,135 17170 Amer, Girona, Spain) during a 13‐day period. The injection of eCG occurred 24 h before sponge removal (400 IU, Bioniche Animal Health (A/Asia), Pty Ltd, Australia). In this experiment, eCG hormone was administered 24 h before sponge removal according to the synchronization protocol routinely applied in Iranian native sheep breeds to enhance follicular recruitment and oestrus synchrony. This timing has been shown in previous regional studies to improve superovulatory response and oestrus synchronization. Notably, the dose of eCG was identical across all treatment groups to ensure that no confounding effects occurred in related to hormone administration (Hemmati et al. 2020; Habibizad et al. 2015). All ewes were checked for signs of oestrus from 12 h after sponge removal, by five intact rams. The ewe was considered to be in oestrus only when she allowed a ram to mount and this was recorded as the time of oestrus onset. Ewes in oestrus, mated twice a day (morning and evening) with Shall rams that were rotates after every four mating. The ewes which did not return to oestrus for an interval equivalent to at least three consecutive oestrus cycles were considered pregnant. The reproductive variables that measured in different groups were: time to onset of oestrus (h), oestrus response (%) and return to oestrus (%).

### 2.3 Evaluation of Follicular Dynamic

To evaluate the effect of treatments on follicular dynamic, the left and right ovaries were examined by daily ultrasonography. Transrectal ultrasonography was conducted from 4 days before to 1 day after sponge removal (Days −4, −3, −2, −1, 0 and +1). Total number, diameter and position of all follicles more than 2 mm were assessed. In each observation, the relative location of all follicles was noted on an ovarian map to follow the sequential follicular development. Then, all visible follicles on the surface of the ovaries were classified based on their size: (a) small (2–3.5 mm), (b) medium (>3.5–5 mm) and (c) large (>5 mm) (Habibizad et al. 2015).

### 2.4 Blood Sampling and Chemical Analysis

Blood samples were taken from jugular vein before the morning feeding (8:00 AM) at the start of experiment and was repeated daily during Days −4 to +1, and also at 12 days after mating. All blood tubes were placed into icy water immediately after collection, and then transported to the laboratory. Then the samples were centrifuged at 2500 rpm for 15 min and serum was separated and stored at −20°C until chemical analysis. Biochemical parameters were selected according to standard studies for the evaluation of metabolic and oxidative responses in ruminants, as previously applied in Choupani et al. (2023) and Jamali Emam Gheise et al. (2017). All kits used for measuring blood metabolites and hormone concentrations were either specifically designed for ruminants or previously validated for use in sheep. The concentration of glucose, triglycerides, cholesterol, total protein, albumin and urea were determined using commercially available kits (Pars Azmoon Company, Tehran, Iran) and an automatic analyser (Alcyon‐300 Auto analyzer; DRG Instruments GmbH, Marburg, Germany). Prior to biochemical analysis, the auto analyser was calibrated using N and P control sera (TrueLab N and TrueLab P Pars Azmoon Co, Iran) and calibration solution) TrueCal U, Pars Azmoon Co, Iran), respectively. Plasma globulins were determined by subtraction albumin from total plasma protein. Moreover, total antioxidant capacity (TAC) (Spectrophotometric kit, Ransel, Randox laboratories ltd, UK and autoanalyzer, Abbott, model Alcyon 300, USA, Intra‐ and inter‐CV < 2.4%), malondialdehyde (MDA) (Based the colourful complex formed from the reaction of MDA with 2‐thiobarbituric acid in an acid environment) and progesterone (Progesterone kit, Diaplus, North York, Canada, Cat No. DP4816, Intra‐ and inter‐CV 5.6% and 13.9%) was measured in serum samples.

### 2.5 Reproductive Performance Assessment

Reproductive traits; including time of birth, gender, birth weight of lamb(s), single births, twin births, triplet births, multiple births (twin births + triplet births), fecundity ([number of lambs born/number of mated ewes] × 100 [number of lambs born/number of ewes lambed] × 100) were calculated following the methods reported by previous researchers (Notter 2008; Macías‐Cruz et al. 2017; Abbott 2024).

### 2.6 Statistical Analysis

This experiment was performed using a completely randomized design with three experimental groups and the GLM procedure of SAS (Version 9.1.3, SAS Institute Inc., Cary, NC). Our model included the fixed effect of treatment and the random effect of ewe within each group. The covariance structure was modelled using the random effect of ewe within groups plus an autoregressive Order 1 to account for the correlation between sequential measurements within the same animal.

Data for response to oestrus, number of lambs born, multiple births, fecundity and prolificacy were analysed using PROC GENMOD. Data whose measurements were repeated over the time (follicular population changes, and concentration of metabolites and hormones) were analysed separately. For the analysis of repeated measurements, the mixed procedure of SAS was used. Mean values were compared by the Duncan's multiple range test. Probability values of less than 0.05 (*p* < 0.05) were considered significant and the tendency toward significance were considered at 0.05 ≤ *p* ≤ 0.1.

## 3 Results

### 3.1 Follicular Characteristic

**FIGURE 1:**
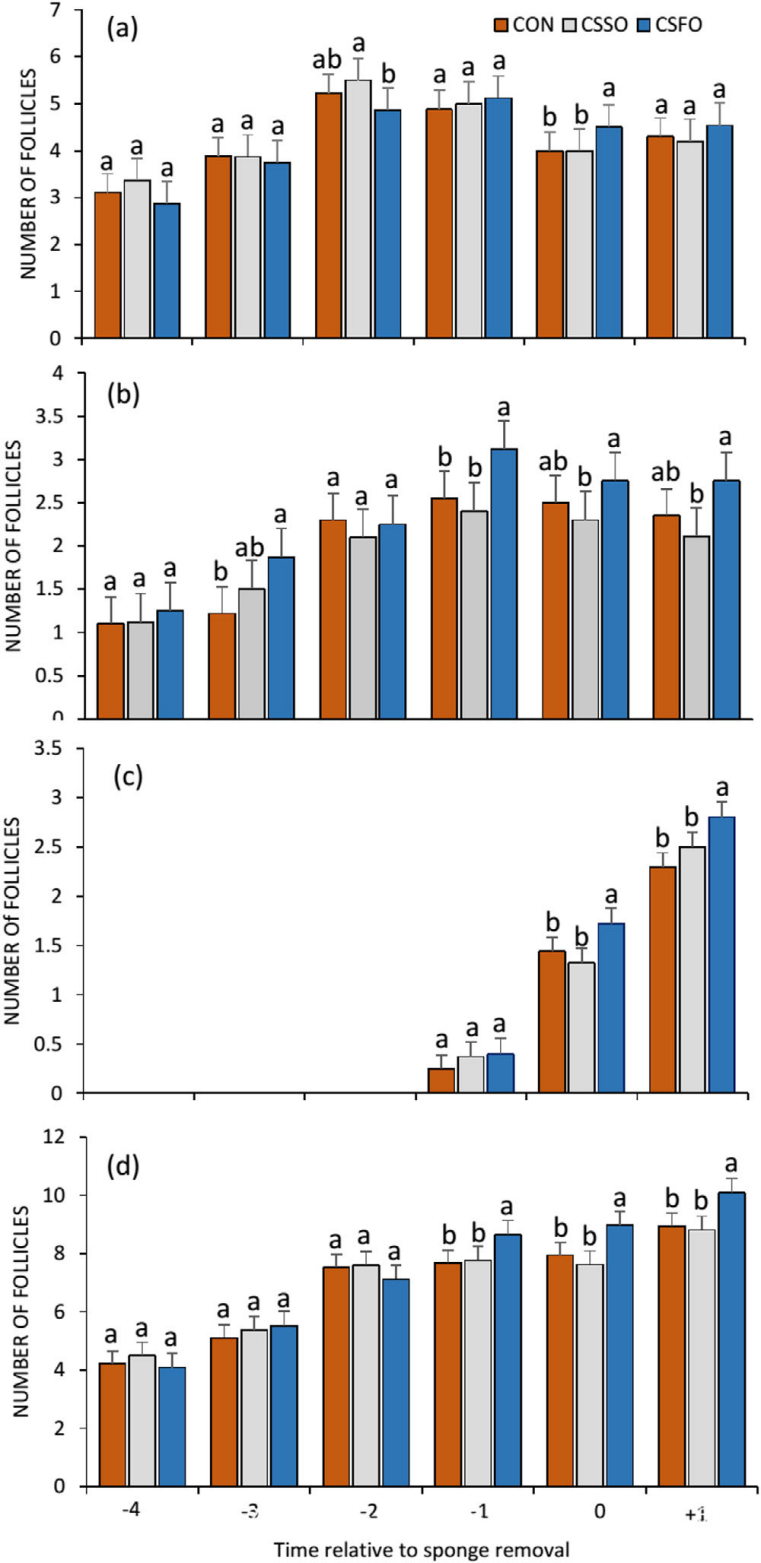
Number of ovarian follicles classified based on the size (a = small; b = medium; c = large and d = total) in Shall ewes. Control diet (CON), diet containing calcium salts of soybean oil (CSSO) and diet containing calcium salts of flaxseed oil (CSFO). Values are expressed as mean ± standard error. The bars with different letters had significant difference (*p* < 0.05).

The populations of small, medium and large size follicles and the total follicles at the different times before and after removing sponge (Days −4 to +1) are presented in Figure 1. In the first days of ultrasonic assessment, no significant difference was observed between the three experimental groups in terms of the number of small and total follicles. One day before sponge removal (Day −1), group CSFO had the highest (*p* < 0.05) number of medium size and total follicles compared to the other groups, but there was no difference (*p* > 0.05) for large size follicles at this time. At the day of sponge removal (Day 0) group CSFO had the highest (*p* < 0.05) number of small and large size and total follicles. On Day +1, the population of small size follicle was not affected by the treatment. However, the large size and total follicles were higher (*p* < 0.05) in CSFO group than those CSSO and CON groups. The ultrasound scanning revealed that the emergence of large‐size follicles occurred on Days −1, 0 and +1 and no large follicles were observed until 1 day before sponge removing.

### 3.2 Oestrus Response

The results of time need to onset of oestrus, oestrus response and return to oestrus are presented in Table 2. Our observation showed that the parameters related to oestrus response was not affected by experimental treatments and all ewes showed oestrus behaviour from 1 to 3 days after sponge removing. The lack of significant differences in time to onset of oestrus, oestrus response and return to oestrus among the experimental groups may be attributed to the limited sample size in treatments.

**TABLE 2:** Effects of dietary treatments on oestrus onset, oestrus response and return to oestrus in Shall ewes.

	Treatments^a^			
Event	CON	CSSO	CSFO	*p*‐value
Time to the onset of estrus (h)	0.91 ± 33.21	1.06 ± 32.87	0.98 ± 34.03	0.7
Oestrus response% (*n*)	100 (9/9)	100 (8/8)	100 (8/8)	1.0
Return to oestrus% (*n*)	0.0 (0/9)	12.5 (1/8)	12.5 (1/8)	0.5

*Note*: Different letters in the same row indicate significant differences (P<0.05).

^a^ Control diet (CON), diet containing calcium salts of soybean oil (CSSO), and diet containing calcium salts of flaxseed oil (CSFO).

### 3.3 Blood Parameters

**FIGURE 2:**
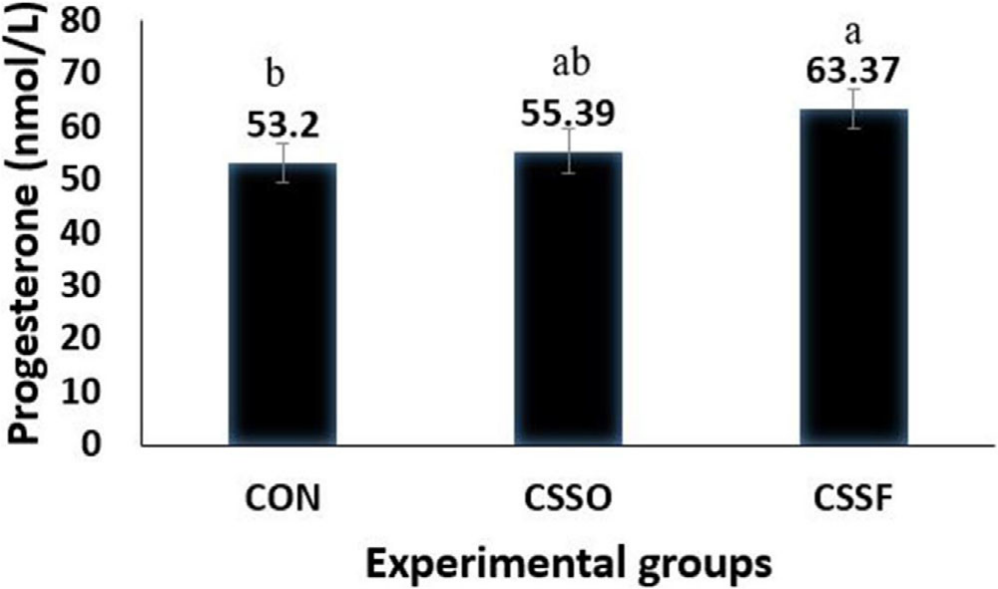
Effect of experimental treatments on serum progesterone concentration in Shall ewes at Day 12 after oestrus. Control diet (CON), diet containing calcium salts of soybean oil (CSSO) and diet containing calcium salts of flaxseed oil (CSFO). Different letters in the same row indicate significant differences (*p* < 0.05).

The results of some blood chemical parameters, and progesterone are presented in Table 3 and Figure 2, respectively. Blood levels of total protein, globulins and urea were not affected by the treatments (*p* > 0.05). On the other hand, blood glucose and TAC tended (*p* = 0.06) to be higher in group CSFO compared to the other groups. Moreover, ewes fed CSFO had lower blood MDA and higher albumin (p < 0.05). Blood triglyceride and cholesterol were affected by source of oil, and feeding CSSO increased (*p* < 0.05) this parameter. Our results showed that 12 days after oestrus, the concentration of blood progesterone was affected by the experimental treatments and it was higher (*p* > 0.05) in CSFO compared to CON groups. The progesterone concentration in ewes fed CSSO was intermediate and had no significant difference with the two other groups.

**TABLE 3:** Effects of experimental dietary treatments on metabolic and oxidative blood parameters in Shall ewes.

	Treatment^a^			*p*‐value		
Blood parameters	CON	CSSO	CSFO	Treat	Day	Treat × Day
Glucose (mmol/L)	3.44 ± 0.05	3.37 ± 0.06	3.51 ± 0.06	0.06	0.11	0.09
Triglycerides (mmol/L)	0.15 ± 0.01b	0.23 ± 0.01a	0.16 ± 0.01b	0.01	0.16	0.88
Cholesterol (mmol/L)	1.67 ± 0.04b	2.08 ± 0.05a	1.85 ± 0.04ab	0.01	0.32	0.76
Total protein (g/L)	62.1 ± 1.30	61.1 ± 0.80	61.5 ± 0.60	0.32	0.22	0.67
Albumin (g/L)	42.3 ± 0.50ab	41.6 ± 0.50b	43.8 ± 0.60a	0.04	0.13	0.42
Globulin (g/L)	19.8 ± 0.70	19.5 ± 0.60	17.7 ± 0.80	0.41	0.21	0.35
Urea (mmol/L)	3.30 ± 0.09	3.52 ± 0.10	3.56 ± 0.09	0.13	0.21	0.51
TAC (mmol/L)	0.31 ± 0.06	0.29 ± 0.07	0.36 ± 0.09	0.06	0.28	0.45
MDA (nmol/mL)	1.55 ± 0.05a	1.59 ± 0.05a	1.47 ± 0.05b	0.05	0.18	0.43

*Note*: Different letters in the same row indicate significant differences (*p* < 0.05).

Abbreviations: HDL, High‐density lipoprotein; TAC, Total antioxidant capacity; MDA, Malondialdehyde.

^a^ Control diet (CON), diet containing calcium salts of soybean oil (CSSO) and diet containing calcium salts of flaxseed oil (CSFO).

### 3.4 Parameters Related to Reproductive Performance

The parameters related to reproductive performance showed in Table 4. Present results revealed that the reproductive variables of ewes were not affected by experimental treatments. However, there were some numerical differences between the different groups. For example, the CON group had higher percent of single and twin births than those the other groups. On the other hand, the triplet birth (42.9%), fecundity (200%) and prolificacy (228.6%) were greater in ewes fed flaxseed oil (CSFO) compared to the other groups. It was surprising that feeding different oils increased the number and percent of female lambs in CSSO and CSFO.

**TABLE 4:** Effects of experimental dietary treatments on lambing and reproductive performance in Shall ewes.

	Treatment^a^			
Parameter	CON	CSSO	CSFO	*p*‐value
Pregnant ewes (%)	9/9 (100)	7/8 (87.5)	7/8 (87.5)	0.54
Single births (%)	2/9 (22.2)	1/7 (14.3)	1/7 (14.3)	0.88
Twin births (%)	6/9 (66.7)	4/7 (57.1)	3/7 (42.9)	0.30
Triplet births (%)	1/9 (11.1)	2/7 (28.6)	3/7 (42.9)	0.35
Multiple births (%)	7/9 (77.7)	6/7 (85.7)	6/7 (85.7)	0.88
Number of lambs born	17	15	16	0.95
Male lambs born	9 (52.9)	5 (33.3)	5 (31.3)	0.66
Female lambs born	8 (47.1)	10 (66.7)	11 (68.8)	0.29
Fecundity (%)	1.88 (188.9)	1.87 (187.5)	2.00 (200.0)	0.65
Prolificacy (%)	1.88 (188.9)	2.14 (214.3)	2.28 (228.6)	0.15

*Note*: Different letters in the same row indicate significant differences (*p* < 0.05). The numbers in parentheses represent the percentage.

^a^ Control diet (CON), diet containing calcium salts of soybean oil (CSSO) and diet containing calcium salts of flaxseed oil (CSFO).

## 4 Discussion

Several studies have shown using PUFA in ruminant diets has potential to affect hypothalamus, pituitary, ovaries and uterus and also change the metabolism and development of follicles (Rahbar et al. 2014; Robinson et al. 2002; Zachut et al. 2010). Results of present study was in line the previous finding, because we revealed that feeding CSFO increased the population of large size and total number of follicles after sponge removal. In agreement with our results, Farrag et al. (2024) showed that feeding non‐pregnant ewes with CSFA enhanced the number of large size follicles. The positive effects of PUFA on follicular dynamic have not been completely elucidated. However, these finding could be related to the change in follicular membrane fluidity by protected unsaturated FAs which facilitates membrane exchanges and therefore better development of follicles (Zachut et al. 2010). Moreover, our observations are in agreement with the findings of Burke et al. (1996) who reported that olive oil and soybean oil had no effect on number of follicles larger than 4 mm in ewes.

Our results showed that dietary treatments had no significant effect on the time to onset of oestrus, oestrus response or return to oestrus. This confirms that the hormonal protocol plays a dominant role in regulating the oestrous cycle, and effects of nutritional interventions, at least under the conditions and duration of this study, were not observed (Santos et al. 2009; Wiltbank et al. 2014). Furthermore, it is possible that longer supplementation periods or larger sample sizes may reveal more evident nutritional effects on oestrus behaviour.

In the present study, dietary supplementation did not affect serum total protein, globulins and urea blood concentrations which indicating minimal influence of PUFA on protein metabolism. These findings in line with previous studies in ruminants, who reported limited effects of fat supplementation on serum protein profiles (Jenkins and Palmquist 1984). Differences in serum glucose levels among treatments were not statistically significant, but it is demonstrated that cows fed CSFO tended to have higher glucose concentrations compared to other groups. This finding suggests a potential improvement in energy status, possibly mediated by enhanced ruminal fermentation or increased intestinal absorption associated with omega‐3 FAs (Mashek and Grummer 2003). The variations in glucose response to dietary fat have been reported, likely reflecting differences in physiological state, energy balance and FA composition across studies (Shingfield and Griinari 2007). Regarding lipid metabolism, CSSO supplementation significantly increased serum triglyceride and cholesterol concentrations relative to the CON and CSFO groups. This is likely attributable to the hyperlipidemic effect of saturated FAs such as palmitic acid, which is present in greater amounts in soybean oil compared to flaxseed oil (El‐Nakhlawy and Shiboob 2011; Murru et al. 2022). Both fat‐supplemented diets elevated serum cholesterol compared to the CON diet and consistent with earlier studies which indicated dietary fat enhances plasma lipid fractions in ruminants (Palmquist and Jenkins 1980). It is well defined that triglycerides play a critical role as an energy source for oocyte maturation and early embryonic development (Ferguson and Leese 2006), emphasizing the reproductive relevance of these metabolic changes. Antioxidant status showed a tendency for improvement in CSFO‐fed cows, reflected by higher TAC and lower MDA concentrations. MDA is a primary product of polyunsaturated FA oxidation and serves as a reliable biomarker for lipid peroxidation (Del Rio et al. 2005; Kotsampasi et al. 2024). In agreement with our results, Bodas and Richardson (2011) reported that supplementing lamb diets with fish oil (rich in n‐3 FAs) reduced plasma lipid peroxidation and improved antioxidant status, indicating enhanced metabolic and immune function. Elevated oxidative stress has been linked to impaired oocyte competence, reduced fertilization rates and compromised embryo quality (Agarwal et al. 2005). Therefore, even moderate improvements in oxidative balance may have positively impact on reproductive performance, particularly under conditions associated with metabolic or heat stress. The most notable reproductive effect observed in this study was the higher serum progesterone concentration on Day 12 post‐oestrus in CSFO‐supplemented sheep which indicates an improvement in luteal function. This finding is consistent with previous studies who reported that supplementation with calcium salts of flaxseed oil and other PUFA increased the progesterone secretion in ruminants (Mahla et al. 2023; Roskopf et al. 2025). Considering that cholesterol is the primary precursor for progesterone synthesis (Miller and Auchus 2011), the elevated serum cholesterol in fat‐supplemented cows may have contributed to enhanced luteal activity. In addition, omega‐3 FAs may exert luteoprotective effects by modulating PGF_2_α synthesis and reducing the likelihood of premature luteolysis (Butler 2003). Overall, the findings from this study suggest that dietary supplementation with calcium salts of FAs, particularly those rich in omega‐3 FAs such as CSFO, positively modulates metabolic, antioxidant and reproductive parameters in sheep.

The findings of the present study indicated that dietary supplementation with CSSO and CSFO had no effect on reproductive parameters. This is likely due to the limited sample size and relatively short supplementation period. However, some numerical trends were observed. Ewes in the CSFO group exhibited higher triplet birth rates and increased fecundity and prolificacy compared with the CON group. These results are consistent with previous studies reporting that dietary fat supplementation, particularly sources rich in PUFAs, may influence reproductive efficiency in small ruminants by improving follicular development and ovulation rates (Akhtar et al. 2024; Mazareei et al. 2024). It is believed that the PUFAs play a key role in reproductive physiology through their involvement in the synthesis of prostaglandins, which regulate follicular dynamics and luteal function. Omega‐6 FAs are precursors of arachidonic acid, which enhances PGF_2_α synthesis, potentially accelerating ovulation and oestrus onset (Mattos et al. 2000). Conversely, omega‐3 FAs can modulate prostaglandin synthesis by reducing PGF_2_α secretion, thereby promoting luteal maintenance and supporting embryo survival (Mahla et al. 2023). This dual mechanism may explain the numerical improvements in reproductive indices observed in the present study. Although the differences in number and sex ratio were not statistically significant, but ewes receiving PUFA‐enriched diets (CSSO and CSFO) produced a higher proportion of female lambs compared to the CON group. Similar observations have been reported by El‐Tarabany et al. (2020), who found that linseed oil supplementation prior to mating increased the proportion of female offspring to nearly 80%. It has been hypothesized that these shifts in sex ratio could be related to changes in follicular fluid composition, hormonal balance and epigenetic regulation of embryonic development (Ngcobo et al. 2023). Furthermore, enhanced uterine receptivity and improved energy balance associated with PUFA intake may contribute to better embryo survival and increased prolificacy.

## 5 Conclusion

Supplementation with calcium salts of soybean or flaxseed oil during the flushing period had no effect on oestrus response or reproductive performance; however, flaxseed oil (CSFO) improved follicular dynamics, enhanced antioxidant status and tended to elevate serum progesterone concentrations. These results indicating the potential benefits on omega 3 FAs for ovarian function and luteal activity. Our findings suggest that while short‐term dietary fat supplementation may not alter oestrus outcomes, it could influence metabolic and endocrine factors which support reproductive efficiency. Future studies with extended supplementation periods and larger sample sizes may warranty and more clarify the long‐term reproductive and physiological benefits of PUFA‐rich diets in ewes.

## Author Contributions

**Mahmood Zeraatkar**: project administration, statistical analysis. **Ahmad Riasi**: conceptualization, project supervision, scientific review, writing – review and editing. **Mohammad Ali Edriss**: project supervision, scientific review. **Javad Habibizad**: project supervision, consultation, scientific review. **Kimia Kazemi**: manuscript proofreading and editing. **Mohammad Choupani**: data analysis and interpretation, writing– original draft, writing – review and editing.

## Funding

The authors have nothing to report.

## Ethics Statement

All experimental procedures involving animals were approved by the Animal Ethics Committee of Isfahan University of Technology, Iran.

## Conflicts of Interest

The authors declare no conflicts of interest.

## Data Availability

The data that support the findings of this study are available from the corresponding author upon reasonable request.
